# Comparative and Functional Analysis of miRNAs and mRNAs Involved in Muscle Fiber Hypertrophy of Juvenile and Adult Goats

**DOI:** 10.3390/genes14020315

**Published:** 2023-01-25

**Authors:** Sanbao Zhang, Qiongwen Zhang, Lili Yang, Xiaotong Gao, Ting Chen, Tianbao Li, Wenyue Sun, Yufan Liu, Zihua Zheng, Yan Pan, Yingming Wei, Yanna Huang, Mingsheng Jiang, Qinyang Jiang

**Affiliations:** 1 College of Animal Science and Technology, Guangxi University, Nanning 530004, China; 2 Institute for New Rural Development, Guangxi University, Nanning 530004, China; 3 College of Animal Science and Technology, Guangxi Agricultural Vocational University, Nanning 530007, China

**Keywords:** miRNA, mRNA, muscle fiber, hypertrophy, goat

## Abstract

MicroRNAs (miRNAs) are small non-coding RNAs that post-transcriptionally regulate several pathway intermediates and affect the skeletal muscle development in mice, pigs, sheep, and cattle. However, to date, only a small number of miRNAs have been reported in the muscle development of goats. In this report, the *longissimus dorsi* transcripts of one- and ten-month-old goats were analyzed by sequencing RNAs and miRNAs. The results showed that the ten-month-old Longlin goats had 327 up- and 419 down-regulated differentially expressed genes (DEGs) compared with the one-month-old. In addition, 20 co-up-regulated and 55 co-down-regulated miRNAs involved in the muscle fiber hypertrophy of goats were identified in ten-month-old Longlin and Nubian goats compared with one-month-old. Five miRNA–mRNA pairs (*chi-let-7b-3p-MIRLET7A*, *chi-miR193b-3p-MMP14*, *chi-miR-355-5p-DGAT2*, *novel_128-LOC102178119*, *novel_140-SOD3*) involved in the goat skeletal muscle development were identified by miRNA–mRNA negative correlation network analysis. Our results provided new insight into the functional roles of goat muscle-associated miRNAs, allowing a deeper understanding of the transformation of miRNA roles during mammalian muscle development.

## 1. Introduction

As a critical agricultural economic animal, the goat (*Capra hircus*) critically contributes to meat, milk, and fiber production [1]. Goat meat has no religious restrictions, which is one of the most freely and widely consumed red meats in parts of Africa, the Middle East, Asia, and the Hispanic population across the USA [2]. Compared to meat from other domestic animals, goat meat contains higher protein content but lower cholesterol and fat content, and it has unique flavors and solid nutritional value [3], garnering increasing attention as a viable source of meat. The *longissimus dorsi* is the most significant part of the ridge of the spine that it has high economic relevance for fresh meat production [4]. It is composed of terminally differentiated multinuclear, postmitotic myofibers [5]. The number and volume of muscle fibers directly affect the skeletal muscle development [6]. However, the number of muscle fibers remains constant after birth [7]. After the birth of mammals, skeletal muscle tissue growth mainly depends on the circumference and length of the muscle fibers [8]. Understanding molecular mechanisms underlying skeletal muscle growth and development is critical for future commercial meat outputs, especially the volume of myofibers. Genetic and environmental factors control skeletal muscle growth and development. 

MiRNAs are single-stranded and non-coding RNAs consisting of 21–25 nucleotides (nts). They form a class of endogenous regulatory factors implicated in structural gene expression, primarily at the post-transcriptional level. The miRNAs regulate essential cellular processes, including cell proliferation, differentiation, and apoptosis [9]. Many reports have identified how miRNAs involve in skeletal muscle genesis, myoblast proliferation, differentiation, and apoptosis in vivo and in vitro. For example, *miRNA-27b-3p* regulate myoblast proliferation and differentiation in chicken by targeting the myostatin gene [10]. *MiR-543* targets *KLF* to regulate myoblast proliferation and differentiation of C2C12 cells [11]. *MiRNA-152* targets *UCP3* to promote slow-twitch myofiber formation in pigs [12]. *MiR-1* initiates muscle development by inhibiting the expression of *HDAC4* in goats [13]. *MiR-365-3p* inhibits proliferation but promotes the differentiation of bovine myoblasts [14]. MiRNAs are essential for analyzing genetic mechanisms underlying skeletal muscle growth and development in pigs, chickens, sheep, and cattle.

Nubian goats, a popular commercialized breed, exhibit high feed efficiency and a fast growth rate, which are common in the hot and humid environment of South China [15]. In the past decades, high meat yields have led to the continuous import of Nubian goats into China, widely used to improve the meat yield of other goat breeds. The Longlin goat is reared in *Guangxi Zhuang Autonomous Region* in southern China. Its unique characteristics include rapid growth and delicious taste, the slight smell of mutton, and excellent meat-producing efficiency [16]. Nubian and Longlin goats share the characteristic of rapid growth; Longlin represents local varieties while Nubian represents introduced breeds. Their bodies can reach maturity at ten months, making them suitable for studying muscle fiber hypertrophy.

This study conducted a high-throughput sequencing strategy to identify *longissimus dorsi* muscle fiber hypertrophy-related mRNAs and miRNAs among the Nubian and Lonlin goats. These findings revealed the molecular underpinnings of miRNAs and the underlying mechanisms operating at different stages of goat skeletal muscle development to provide novel insights for molecular goat breeding.

## 2. Materials and Methods

This study followed strictly the animal ethical standards and regulations of the College of Animal Science and Technology, Guangxi University (approval number 2021-001). 

### 2.1. Experimental Animals and Sample Collection

Twelve healthy rams were selected for the investigation at the *Guangxi Lvshijie Agricultural investment co. Ltd. in Nanning County, Guangxi Zhuang Autonomous Region, China,* including six Nubian rams (three one-month-old and three ten-month-old) and six Longlin rams (three one-month-old and three ten-month-old). They were fasted for 24 h and then slaughtered. The *longissimus dorsi* were collected as experimental samples. After collection, the samples were subjected to three PBS washes. One group of samples was snap-frozen in liquid nitrogen and then stored at −80 °C before RNA isolation. In addition, the *longissimus dorsi* mass (1 cm × 1 cm × 1.5 cm) was quickly put into the constant cold slicer, sliced at −20 °C, with a thickness of 8 mm. The slices were dried at room temperature for 30 min, followed by hematoxylin-eosin (H&E) staining and Adenosine Triphosphatase (ATPase) Staining. 

### 2.2. Paraffin Section and H&E Staining

H&E staining was performed by the conventional staining method. Briefly, with about 4 min of hematoxylin staining followed by 0.5% eosin staining for 3 min. Images were acquired by an inverted fluorescent microscope (ZEISS, Germany) with 20× objective lens (200× magnification), and the skeletal muscle fiber diameter was measured by Image J software (National Institutes of Health, Bethesda, MD, USA). 

### 2.3. ATPase Staining 

After the drying of frozen sections were labeled with a histochemical pen, and a pH 10.4 and 9.4 incubation solution was added and incubated for 5 min and 30 min subsequently at room temperature, respectively. After pouring off the incubation solution, the sections were directly soaked into CaCL2 solutions for 2 min. Then, the sections were directly immersed in cobalt nitrate solution for 5 min and drip dyeing 1% ammonium sulfide solution for 30 s. Images were acquired by inverted fluorescent microscope (ZEISS, Germany) with 20× objective lens (200× magnification) after cleaning by water for 20 s, and the skeletal muscle fiber diameter was measured by Image J software (National Institutes of Health, Bethesda, MD, USA). 

### 2.4. RNA Isolation, Library Preparation, and Sequencing

Total RNA was extracted using the TRIzol Reagent (Invitrogen Life Technologies, Carlsbad, CA, USA) according to the manufacturer’s instructions. Then, mRNA and miRNA libraries construction by TruSeq Stranded mRNA LT sample preparation kit (Illumina, San Diego, CA, USA) and TruSeq Small RNA Sample Prep Kit Illumina (San Diego, CA, USA), respectively. Novogene Bioinformatics Institute (Novogene, Beijing, China) performed the miRNA and mRNA omics-based sequencing. 

### 2.5. Sequencing Quality Control and Expression Analysis

Quality control of all the raw sequencing reads was controlled by FastQC (https://www.bioinformatics.babraham.ac.uk/projects/fastqc/ (accessed on 13 August 2022)). Then, the clean reads were aligned to the goat genome (*Capra hircus ARS1.2*) [17] by HISAT2 (v2.2.1). The rRNAs, tRNAs, snRNAs, and snoRNAs in the matched sequences were filtered out by Rfam (https://rfam.sanger.ac.uk/ (accessed on 22 August 2022)). Then, conserved miRNAs were identified by the remaining reads were aligned to the miRNA precursors in goats of miRBase database. Further, candidate novel miRNAs were predicted by the unannotated sRNA reads, which were annotated with MIREAP software (http://sourceforge.net/projects/mireap (accessed on 27 August 2022)). The miRNA expression was normalized by transcripts per million (TPM) method: normalized expression = (actual miRNA sequencing read count/total miRNA reads count) × 1,000,000. 

### 2.6. Analysis of DEMs and DEGs 

Differential expression analysis of mRNAs and miRNAs was utilized by a DESeq2 R package (version 3.14.0) [18] with an absolute value of log_2_ fold change (log_2_FC) > 1.0 and *p*-value < 0.05.

### 2.7. Target Gene Prediction for DEMs

PITA, miRanda, and RNAhybrid software intersecting were used to predict miRNA–mRNA interaction networks to better understand how miRNAs bind to mRNAs. Briefly, the target gene prediction was performed through the combination of Miranda (http://www.microrna.org/microrna/ (accessed on 5 September 2022)) using a score ≥ 150, energy ≤ 20, and RNAhybrid with energy ≤ 25.

### 2.8. Gene Oncology and KEGG Analyses

The present study used hypergeometric distribution to screen for significantly enriched Gene Ontology (GO) terms and Kyoto Encyclopedia of Genes and Genomes (KEGG) pathways by the DEGs and DEMs target genes. GO is an international standard classification system for gene function (http://www.geneontology.org/ (accessed on 13 September 2022)) and can be divided into three major components: molecular function, cellular component, and biological process. KEGG is the central public database that curates pathways involved in biological systems. The most important metabolic pathways and signaling pathways related to the DEGs were determined by analyzing the significant enrichment pathways and applying a hypergeometric test. This analysis was necessary to further understand the biological function of the genes of interest.

### 2.9. Quantitative Reverse Transcription PCR (RT-qPCR)

Total RNA was extracted from *longissimus dorsi* using RNAiso Plus (Takara, Japan) according to the manufacturer’s instructions. For cDNA synthesis, 2 μg of RNA was used through reverse transcription using PrimeScript RT Master Mix (Takara, Japan). Quantitative real-time polymerase chain reaction was performed in triplicate in a 96-well plate using 1 μl of cDNA and SYBR Green PCR mix (Bio-Rad) on a CFX96 Real-Time PCR System (Takara, Japan). The primer sequences of these miRNAs are listed (Table S5). Expression of beta-actin was used to normalize gene expression. By convention, changes in expression were determined using 2^−ΔΔct^ method.

### 2.10. Statistical Analysis

Data from the study were subjected to one-way analysis of variance using SPSS 20.0, and significant means were compared using Duncan’s multiple range tests of the same software at 5% significance level. Results are presented as mean ± standard error of mean.

## 3. Results

### 3.1. Muscle Fiber Enlargement-Based Characterization of the Skeletal Muscle of Adult Goats

**Figure 1:**
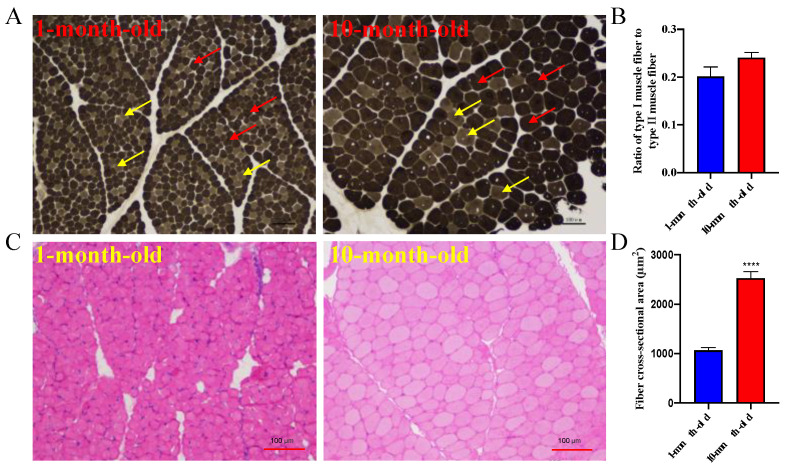
The characteristic of muscle fibers for *longissimus dorsi* between 10-month-old and 1-month-old Longlin goat. (**A**) ATPase staining of *longissimus dorsi* between 10-month-old and 1-month-old Longlin goat. Type I is light gray or colorless (yellow arrow), type II is dark gray or black (red arrow). (**B**) Statistics of fiber types for *longissimus dorsi* between 10-month-old and 1-month-old Longlin goat. (**C**) H&E staining of *longissimus dorsi* between 10-month-old and 1-month-old Longlin goat. (**D**) Statistics of fiber cross-sectional area for *longissimus dorsi* between 10-month-old and 1-month-old Longlin goat. H&E staining: hematoxylin-eosin staining; ATPase Staining: Adenosine Triphosphatase Staining.

ATPase and H&E staining were performed on the *longissimus dorsi* of ten-month-old and one-month-old Longlin goats to observe the changes in muscle fiber types during the development of goat skeletal muscle, which revealed no significant differences (*p* = 0.0810) (Figure 1A,B). However, the average diameters of fibers for the *longissimus dorsi* of a ten-month-old were significantly bigger than a one-month-old Longlin goat (Figure 1C).

### 3.2. Global mRNA Expression Patterns of Longissimus Dorsi of Longlin Goat

**Figure 2:**
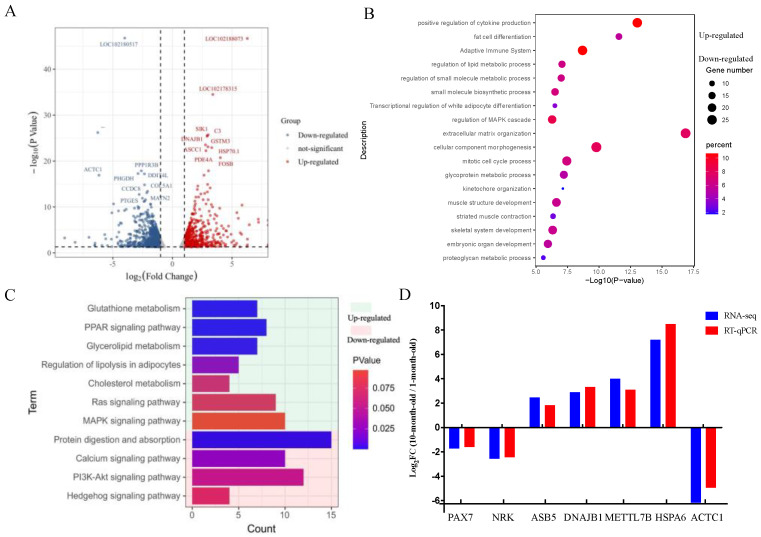
Differential expression analysis of mRNAs in *longissimus dorsi* between 1-month-old and 10-month-old Longlin goat. (**A**) Volcano plots of up-regulated and down-regulated DEGs of *longissimus dorsi* between 10-month-old and 1-month-old Longlin goat. The gray dots represent non-significantly DEGs, the 327 red dots represent significantly differentially up-regulated mRNAs, and the 419 blue dots represent significantly differentially down-regulated mRNAs. (**B**) GO analysis of DEGs of *longissimus dorsi* between 10-month-old and 1-month-old Longlin goat. Light green represents terms of up-regulated gene; Light red represents terms of down-regulated gene. (**C**) KEGG analysis of DEGs of *longissimus dorsi* between 10-month-old and 1-month-old Longlin goat. Light green represents terms of up-regulated genes; Light red represents terms of down-regulated genes. (**D**) RT-qPCR of DEGs compared with RNA-seq. Results of RT-qPCR analysis (Parallel analysis with RNA-seq results) of DEGs fold-change in *longissimus dorsi* between 10-month-old and 1-month-old Longlin goat. The relative expression of each mRNA was calculated using the 2^−ΔΔct^ method. The data are presented as mean ± SEM from three independent experiments.

We then analyzed transcriptomic differences by RNA-seq to investigate mRNA expression changes of *longissimus dorsi* between ten-month-old and one-month-old Longlin goats. The quality assessment of the sequencing data was summarized in Table S1. Then, the clean reads were mapped to the *Capra hircus* genome (Table S2). A heat map of Pearson correlation between samples showed the cluster analysis of the one- and ten-month-old goats (Figure S1) and indicated the reproducibility of sequencing samples are high. Genes were defined as differentially expressed (DE) when the *p* value was <0.05 (p_adj_ < 0.05) and |log_2_foldchange| > 1.0. As shown in Figure 2A, the ten-month-old Longlin goat expressed 746 DEGs (327 up- and 419 down-regulated DEGs) compared with the one-month-old group. 

GO and KEGG were used to explore the biological functions of all DEGs. Representative enriched GO terms and KEGG enrichment pathways for DEGs are shown in Figure 2B,C. The functions enriched for up-regulated genes included “fat cell differentiation”, “regulation of lipid metabolic process”, “regulation of small molecule metabolic process”, “small molecule biosynthetic process”, “transcriptional regulation of white adipocyte differentiation” terms, and “*PPAR* signaling pathway (e.g., *ADIPOQ*, *PPARD*, *FABP5*)” and “regulation of lipolysis in adipocytes (e.g., *CIDEC*, *ADRB2*, *PLIN1*)” pathways. Further, the downregulated genes showed the enrichment of the “mitotic cell cycle process”, “muscle structure development”, “striated muscle contraction”, “skeletal system development” terms, and “PI3K-Akt signaling pathway (e.g., *FGFR4*, *IGF2*, *CHAD*)” and “Apelin signaling pathway (e.g., *NOTCH3*, *MYL4*, *APLNR*)” pathways. These results implied that the process of skeletal muscle development from one- to ten-month-old will be accompanied by lipid metabolism, weakening the proliferation ability of skeletal muscle cells.

Next, seven randomly selected mRNAs were measured by RT-qPCR. Comparison of RT-qPCR and RNA-Seq data showed that they have consistent expression trends (Figure 2D) and indicating that the RNA-seq data are reflective of actual changes at the genetic level.

### 3.3. Overview of miRNA Expression Patterns of Longissimus Dorsi of Longlin and Nubian Goats

**Figure 3:**
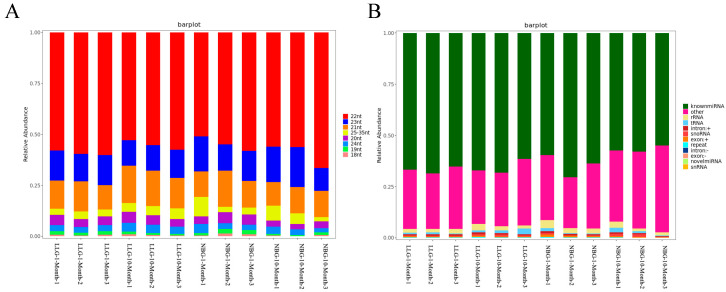
miRNA expression profiles in *longissimus dorsi* of goat. (**A**) Percentage distribution of all small RNA sequence lengths in *longissimus dorsi* of goat. (**B**) Characteristics of known and novel miRNAs in *longissimus dorsi* of goat. Small RNA classification was performed using the priority of “known miRNA > rRNA > tRNA > snRNA > snoRNA > repeat > gene > novel miRNA”.

We constructed 12 small RNA libraries for six domestic native goat breeds (Longlin goat) and six introduced goat breeds (Nubian goat) to obtain the miRNA expression characteristic related to muscle fiber hypertrophy of *Longissimus dorsi*. The libraries were generated in 12 small RNA libraries ranging from 11793428 to 23368197, with an average of 16766641 raw reads. Then, removing adaptor sequences and low-quality reads to obtain clean reads from each library, average 16429930 (Table S3), and successfully mapped to the reference genome sequence of *Capra hircus*. The percentage of goat clean reads in each RNA-seq library is more than 95%. The length distribution of all clean reads was summarized, and they found that in 12 samples, the length of most clean reads obtained were 22–24 nts, with 22 nts lengths being the most common (Figure 3A), previous corroborating findings.

MiRNAs represent the percentage of nucleic acids in Figure 3B; 92.15–96.16% of the sRNA was mapped to the reference sequence of sRNA libraries (Table S4). Subsequently, the annotated sRNAs varying from 18 to 35 nts were divided into several categories: known miRNA (average 63.43%), intro (+ and-link, average 0.74%), exon (+ and-link, average 0.56%), rRNA (average 2.26%), snoRNA (average 0.57%), novel miRNA (average 0.02%), snRNA (average 0.02%), and others (average 31.15%). The known miRNAs accounted is the highest among them. BLASTN analysis with *Capra hircus* miRNAs by miRBase 20.0 showed that 410 known and 90 novel goat miRNAs were identified (Table S4). Because of the specificity of the cleavage site, the first base of the miRNA mature sequence had a strong bias. Our results showed that the primary base preference was for U, but miRNAs of 26 nt lengths highly preferred A. A heat map of Pearson correlation between samples showed the cluster analysis of the one-month-old and ten-month-old Nubian and Longlin goats (Figure S2). These data validated the experiment’s reliability, which showed a high correlation within samples at each stage, confirming the reliability between the samples at the same stage. 

### 3.4. Differential Levels of miRNAs Expressed in Different Breeds and Age of Goats

**Figure 4:**
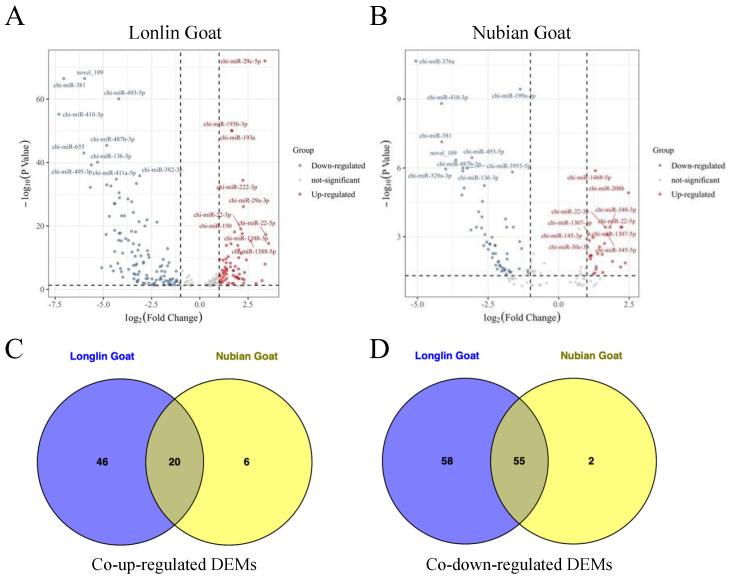
Identification of DEMs between 1-month-old and 10-month-old Longlin and Nubian goat. (**A**) Volcano plots of up-regulated and down-regulated DEMs of *longissimus dorsi* between 10-month-old and 1-month-old Longlin goat. The gray dots represent non-significantly DEMs, the 66 red dots represent significantly differentially up-regulated miRNAs, and the 113 blue dots represent significantly differentially down-regulated miRNAs. (**B**) Volcano plots of up-regulated and down-regulated DEMs of *longissimus dorsi* between 10-month-old and 1-month-old Nubian goat. The gray dots represent non-significantly DEMs, the 26 red dots represent significantly differentially up-regulated miRNAs, and the 57 blue dots represent significantly differentially down-regulated miRNAs. (**C**) Venn diagram showing the intersection of the co-up-regulated DEMs between Longlin and Nubian goat. (**D**) Venn diagram showing the intersection of the co-down-regulated DEMs between Longlin and Nubian goat.

We then used TPM to normalize expression levels of known and novel miRNAs by detecting them by the density distribution of TPM in each sample. Based on the volcano plots, among which 179 significant DEMs in ten-month-old Longlin goat (including 66 up-regulated and 113 down-regulated miRNAs) were identified compared with one-month-old Longlin goat (Figure 4A) and 83 significant DEMs in ten-month-old Nubian goat (including 26 up-regulated and 57 down-regulated miRNAs) compared with one-month-old Nubian goat (Figure 4B, Table S6). We also compared the common DEMs of Chinese natives (Longlin goat) and introduced breeds (Nubian goat) at different stages. Seventy-five overlapped DEMs (including 20 co-up-regulated and 55 co-down-regulated DEMs) were founded (Figure 4C,D). 

### 3.5. Target Prediction for DEMs, GO, and KEGG Analyses

The miRNA regulates biological processes by binding and interacting with targets. RNAhybrid, PITA, and miRanda software were used to predict target genes for both known and novel miRNAs; 183 and 107 target genes were predicted by co-up-regulated DEMs and co-down-regulated DEMs, respectively. Many DE miRNA–mRNA pairs were identified, and it was found that one mRNA can be targeted and regulated by multiple miRNAs. In contrast, one miRNA can regulate many mRNAs simultaneously.

**Figure 5:**
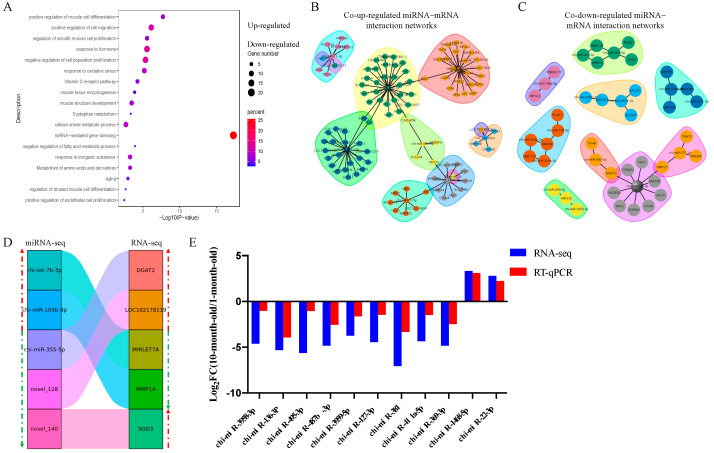
Function enrichment and joint analysis of DEMs and DEGs. (**A**) Function enrichment analysis of common DEM target genes in *longissimus dorsi* of goat. Light green represents terms of up-regulated miRNA target genes; Light red represents terms of down-regulated miRNA target genes. (**B**) The co-up-regulated miRNAs and target genes interaction networks in *longissimus dorsi* of goat. (**C**) The co-down-regulated miRNAs and target genes interaction networks in *longissimus dorsi* of goat. (**D**) miRNA–mRNA negative correlation network in *longissimus dorsi* of goat. The green arrow represents the down-regulated genes and miRNAs, and the red arrow represents up-regulated genes and miRNAs. (**E**) RT-qPCR of DEMs compared with RNA-seq. Results of RT-qPCR analysis (Parallel analysis with RNA-seq results) of DEMs fold-change in *longissimus dorsi* between Longlin. The relative expression of each miRNA was calculated using the 2^−ΔΔct^ method. The data are presented as mean ± SEM from three independent experiments.

The GO annotation and KEGG pathway analysis were performed to identify functional modules of the DEM target genes by Metascape (http://metascape.org/gp/index.html#/main/step1 (accessed on 23 September 2022)). Terms of “positive regulation of muscle cell differentiation”, “positive regulation of cell migration”, “regulation of smooth muscle cell proliferation”, “response to the hormone”, “negative regulation of cell population proliferation”, “muscle tissue morphogenesis”, and “muscle structure development” were enriched for co-up-regulated DEM target genes. Terms including “negative regulation of the fatty acid metabolic process”, “metabolism of amino acids and derivatives”, “aging”, “regulation of striated muscle cell differentiation”, and “positive regulation of endothelial cell proliferation” were enriched for co-up-regulated DEM target genes (Figure 5A). Interestingly, these target genes did not show enrichment for terms related to muscle growth and development, which may be caused by the relatively few target genes we predicted and the strict prediction conditions.

Further, a joint analysis of the interactions between DE miRNA–mRNA pairs with a negative correlation was performed (Figure 5B–D). Compared with a one-month-old goat, the expression of *chi-let-7b-3p* and *chi-miR-193b-3p* were significantly up-regulated at ten-month, and the expression of *chi-miR-355-5p*, *novel_128*, and *novel_140* was significantly down-regulated at ten-month, identified by miRNA-seq. However, the expression of the corresponding target genes (*DGAT2*, *LOC102178139*, *MIRLET7A*, *MMP14*, *SOD3*) showed opposite regulation in the RNA-seq of the Longlin goat. These results implied that the interactions between miRNA and mRNA might be involved in muscle fiber hypertrophy. 

In our analysis, *chi-miR-381, chi-miR-487b-3p, chi-miR-495-3p, chi-miR-136-3p*, and *chi-miR-369-3p* are the five highest DEM. Then, we randomly selected six other DEMs and validated their expression patterns using RT-qPCR. The results showed that their expression patterns agreed with those from RNA-seq analysis (Figure 5E) and indicated the validity of the RNA-seq data.

## 4. Discussion

In recent years, with the development of the national economic level, consumers have been more inclined towards mutton because of its juiciness, functional nutrients, and flavor. Thus, improving the meat yield index becomes an essential part of the breeding of goats. In previous studies, researchers were more interested in muscle development and growth, which involved the expression of some genes, gene polymorphism, and so on. In contrast, some studies focused on the gene expression profiling of skeletal muscle. However, limited information on the expression profiling of miRNAs during the myogenesis of goats is available, and it is inconclusive. In the present study, we selected Longlin and Nubian goats as the representative breeds to detect the differential expression of critical mRNAs and miRNAs in *longissimus dorsi* at different growth stages using high-throughput sequencing specific to muscle development.

Muscle growth involves increased muscle fiber number (hyperplasia) and muscle fiber size (hypertrophy) [19]. However, the number of muscle fibers are fixed during embryonic development [20]. We used goats at one and ten months of age as experimental subjects to analyze mRNA and miRNA expression differences to understand the molecular mechanism of goat muscle fiber hypertrophy. RNA-seq results showed that the ten-month-old Longlin goat expressed 327 up- and 419 down-regulated mRNAs compared with the one-month-old Longlin goat. A functional enrichment analysis for up-regulated genes (*PPARD*, *CIDEC*, *LPL*) showed significant enrichment of biological processes and pathways related to lipid metabolism. Further, the downregulated genes (*NOTCH3*, *MYL10*, *MMP14*, *IGF2BP3*) showed enrichment of the muscle structure development processes and pathways. These results collectively implied that the process of skeletal muscle development from one- to ten-month-old will be accompanied by lipid metabolism and weakened skeletal muscle cells’ weakened proliferation ability.

The miRNAs are short, endogenous, non-coding RNAs, 19-22 nts in length [21]. One- and ten-month-old local representative goat varieties (Longlin) and introduced varieties (Nubian) *longissimus dorsi* were selected as subjects to enhance the accuracy and representativeness of goat muscle fiber hypertrophy-related miRNAs. The present study obtained approximately 99% clean reads from the raw reads in each sample, the percentage of high-quality reads was nearly 100%, and most of the clean reads (92.15–96.16%) matched the *Capra hircus* genome, validating the high quality of our sRNA-seq data. Read length distributions of 12 libraries demonstrated that most sRNAs were 20 to 24 nts long, most of which were 22 nts long. Bases were at most U. These findings corroborated the normal size of miRNAs reported in the previous studies [22]. From this analysis, we established the reliability of the data and analyzed the differences. At the same time, the report applied to two representative goat breeds, Chinese native and introduced goats, excluding the breeds’ specificity, which increased the persuasion and reliability of our data. Seventy-five miRNAs involved in muscle fiber hypertrophy were confirmed to be differentially expressed between Chinese native (Longlin goat) and introduced breeds (Nubian goat). For example, *miR-145* activates *IGF11R/PI3K/AKT* pathway and promotes muscle proliferation and differentiation [23], *miR-195* regulates muscle cell proliferation and differentiation [24], and *miR-17* and *miR-19* cooperatively promote skeletal muscle cell differentiation [25]. Meanwhile, the genes targeting the DEMs were predicted, which revealed that *IGFBP5* targets *chi-miR-504*, and *chi-miR-29b-5p* is targeted by *MYOG*. *IGFBP-5* can regulate muscle cell differentiation [26], and *MYOG* is a muscle-specific transcription factor [27]. GO and KEGG enrichment analysis suggested that the genes targeting by DEMs were closely correlated to “positive regulation of muscle cell differentiation” and “muscle structure development”, respectively. Significantly, five miRNA–mRNA pairs (*chi-let-7b-3p-MIRLET7A*, *chi-miR193b-3p-MMP14*, *chi-miR-355-5p-DGAT2*, *novel_128-LOC102178119*, and *novel_140-SOD3*) involved in the goat skeletal muscle hypertrophy were identified by miRNA–mRNA negative correlation network analysis. A previous report showed that *MMP14* is necessary to invade three-dimensional collagen by human muscle satellite cells [28]. *DGAT2* enzymatically catalyzes the final step of triacylglycerol (TG) synthesis and is closely related to carcass and meat quality [29,30]. Our findings collectively provide valuable information about candidate genes associated with skeletal muscle development and highlight their potential role in muscle phenotype variation between goat breeds. The data generated from this study will be informative for building the basis for further muscle development and molecular breeding studies. However, further studies are needed to decipher the biological functions of these DEGs and miRNAs.

## 5. Conclusions

In conclusion, our findings identify candidate genes and miRNAs associated with muscle development and indicate their potential roles in muscle fiber hypertrophy. These findings will bolster further studies on goat muscle development and molecular breeding.

## Data Availability

The data that support the findings of this study have been deposited in the NCBI Gene Expression Omnibus (GEO) Database under Accession Number GSE217092 (https://www.ncbi.nlm.nih.gov/geo/query/acc.cgi?acc=GSE217092). In addition, the datasets used and analyzed during the current study are available from the corresponding author on academic request.

## Supplementary Materials

The following supporting information can be downloaded at: https://www.mdpi.com/article/10.3390/genes14020315/s1, Figure S1: Heat map of replicate samples between 10-month-old and 1-month-old Longlin goat.; Figure S2: Heat map of replicate samples between 1-month-old and 10-month-old Longlin and Nubian goat.; Table S1: Statistical analysis of the mRNAs Libraries; Table S2: Statistical analysis of the mRNAs Libraries. Table S3: Statistical analysis of the small RNAs Libraries; Table S4: Statistical analysis of the Small RNAs Libraries. Table S5: Primers used for RT-qPCR in this study. Table S6: List of co-DE miRNAs between 1-month-old and 10-month-old Longlin and Nubian goat.
