# Supplementary material for: Comparative and Functional Analysis of miRNAs and mRNAs Involved in Muscle Fiber Hypertrophy of Juvenile and Adult Goats
Source: Genes (Basel). 2023 Jan 25;14(2):315. doi: 10.3390/genes14020315 (PMC9956283; doi:10.3390/genes14020315)
Supplement: Supplementary file 1 [file genes-14-00315-s001.zip › genes-2160867-supplementary.pdf]

Comparative and functional analysis of miRNAs and mRNAs involved in muscle fiber hypertrophy of juvenile and adult goats

Sanbao Zhang [1], Qiongwen Zhang [1], Lili Yang [1], Xiaotong Gao [1], Ting Chen [1], Tianbao Li [1], Wenyue Sun [1], Yufan Liu [1], Zihua Zheng [2], Yan Pan [3], Yingming Wei [2], Yanna Huang [1], Mingsheng Jiang [1,*] and Qinyang Jiang [1,*]

[1] College of Animal Science and Technology, Guangxi University, Nanning 530004, China
[2] Institute for New Rural Development, Guangxi University, Nanning 530004, China
[3] College of Animal Science and Technology, Guangxi Agricultural Vocational University, Nanning 530007, China
* Correspondence: 13878896387@163.com (M.J.) and jiangqinyang2013@gxu.edu.cn (Q.J.)

Supplementary Material

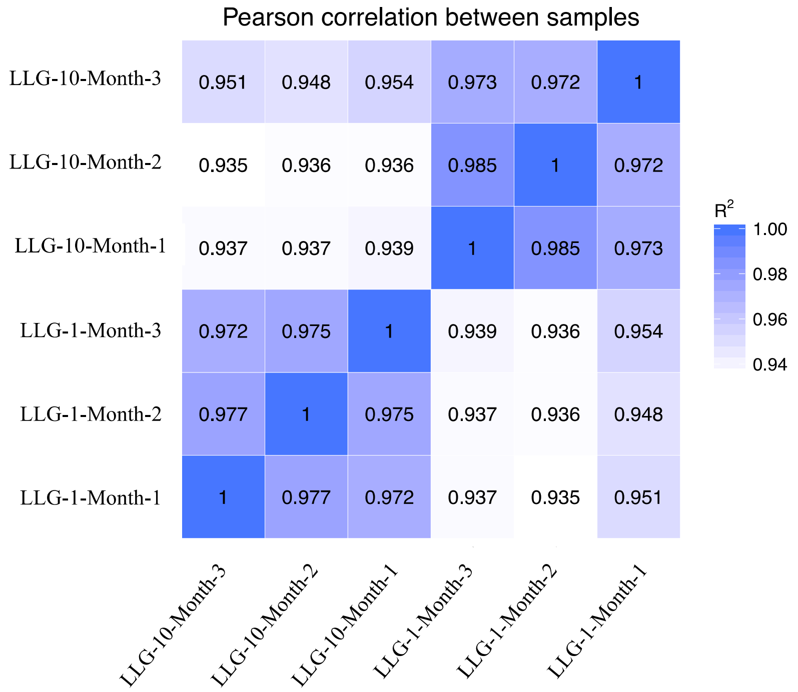

Figure S1 Heat map of replicate samples between 10-month-old and 1-month-old Longlin goat. The color spectrum, ranging from white to blue, represents Pearson correlation coefficients ranging from 1 to 0.75, indicating high to low correlations.

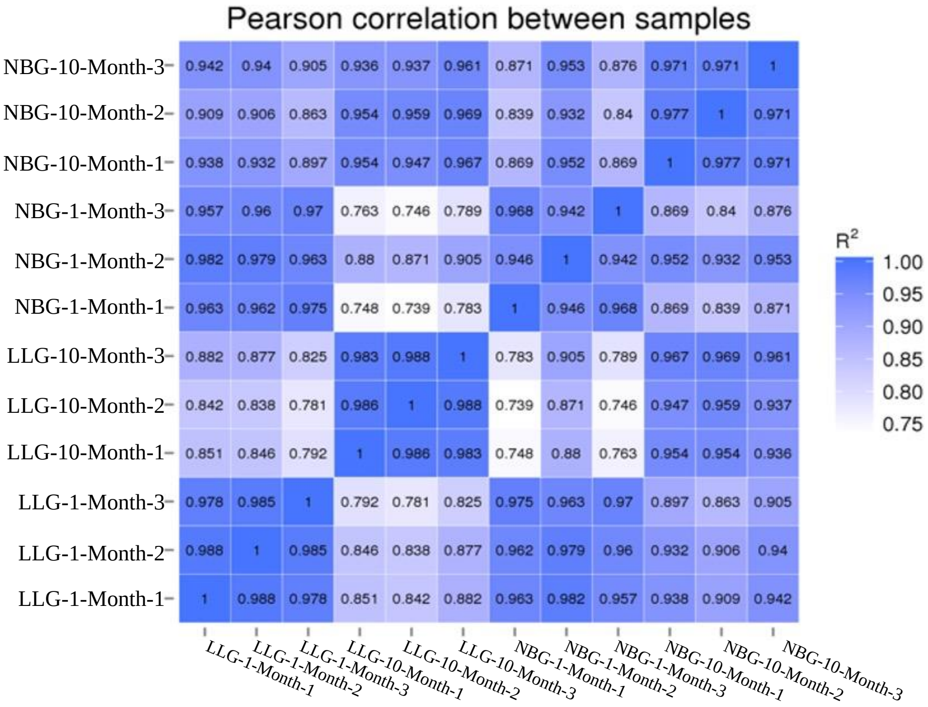

2

Figure S2 Heat map of replicate samples between 1-month-old and 10-month-old Longlin and Nubian goat. The color spectrum, ranging from white to blue, represents Pearson correlation coefficients ranging from 1 to 0.75, indicating high to low correlations.

3

Table S1 Statistical analysis of the mRNAs Libraries.

Sample name	Raw reads	Clean reads	UMI reads	Raw bases	Clean bases	Error rate (%)	Q20 (%)	Q30 (%)	GC content (%)	UMI2Clean (%)	Dedup2MappedUMI (%)	Deduped ratio (%)
LLG-1-Month-1	41644544	40853022	39755516	6.25G	6.13G	0.03	97.47	92.58	52.55	97.31	79.63	20.37
LLG-1-Month-2	43894716	43040954	41898550	6.58G	6.46G	0.03	97.74	93.15	52.35	97.35	76.86	23.14
LLG-1-Month-3	43220282	42498144	41338468	6.48G	6.37G	0.03	97.66	93.02	52.59	97.27	77.77	22.23
LLG-10-Month-1	40942442	39965722	38884046	6.14G	5.99G	0.03	97.68	93.02	53.25	97.29	78.3	21.7
LLG-10-Month-2	39610564	38805376	37704982	5.94G	5.82G	0.03	97.66	93.09	53.27	97.16	78.1	21.9
LLG-10-Month-3	40588140	39784988	38888042	6.09G	5.97G	0.02	98.38	95.15	54.39	97.75	77.69	22.31

Table S2 Statistical analysis of the mRNAs Libraries.

Sample name	LLG-1-Month-1	LLG-1-Month-2	LLG-1-Month-3	LLG-10-Month-1	LLG-10-Month-2	LLG-10-Month-3
UMI reads	39755516	41898550	41338468	38884046	37704982	38888042
UMI mapped	38542870 (96.95%)	40881825 (97.57%)	40110047 (97.03%)	37893289 (97.45%)	36729986 (97.41%)	37936467 (97.55%)
Multiple mapped	3040036 (7.65%)	2684102 (6.41%)	2825633 (6.84%)	2269628 (5.84%)	1864701 (4.95%)	2164564 (5.57%)
Uniquely mapped	35502834 (89.3%)	38197723 (91.17%)	37284414 (90.19%)	35623661 (91.62%)	34865285 (92.47%)	35771903 (91.99%)

Read-1	17837165 (44.87%)	19173785 (45.76%)	18716291 (45.28%)	17896765 (46.03%)	17525013 (46.48%)	17952073 (46.16%)
Read-2	17665669 (44.44%)	19023938 (45.4%)	18568123 (44.92%)	17726896 (45.59%)	17340272 (45.99%)	17819830 (45.82%)
Reads map to "+"	17738845 (44.62%)	19123370 (45.64%)	18623934 (45.05%)	17822399 (45.83%)	17445347 (46.27%)	17894876 (46.02%)
Reads map to "-"	17763989 (44.68%)	19074353 (45.53%)	18660480 (45.14%)	17801262 (45.78%)	17419938 (46.2%)	17877027 (45.97%)
Non-splice reads	18208107 (45.8%)	19882579 (47.45%)	18431003 (44.59%)	17080245 (43.93%)	16865484 (44.73%)	16375230 (42.11%)
Splice reads	17294727 (43.5%)	18315144 (43.71%)	18853411 (45.61%)	18543416 (47.69%)	17999801 (47.74%)	19396673 (49.88%)

Table 3 Statistical analysis of the small RNAs Libraries.

Sample	Reads	Bases	Error rate	Q20	Q30	GC content	N% > 10%	low quality	5 adapter contamine	3 adapter null or insert null	with ployA/T/G/C	clean reads
NBG-1-Month-1	17651855	0.883G	0.01%	99.50%	98.67%	46.98%	245 (0.00%)	13334 (0.08%)	2509 (0.01%)	598914 (3.39%)	20662 (0.12%)	17016191 (96.40%)
NBG-1-Month-2	16608446	0.830G	0.01%	99.52%	98.72%	46.17%	252 (0.00%)	10412 (0.06%)	797 (0.00%)	256778 (1.55%)	8835 (0.05%)	16331372 (98.33%)
NBG-1-Month-3	18325032	0.916G	0.00%	99.78%	99.43%	45.38%	18 (0.00%)	5631 (0.03%)	900 (0.00%)	274201 (1.50%)	9794 (0.05%)	18034488 (98.41%)
NBG-10-Month-1	23368197	1.168G	0.01%	99.57%	98.86%	46.06%	335 (0.00%)	13581 (0.06%)	3068 (0.01%)	753850 (3.23%)	14485 (0.06%)	22582878 (96.64%)
NBG-10-Month-2	19218100	0.961G	0.01%	99.54%	98.80%	45.42%	305 (0.00%)	12620 (0.07%)	3201 (0.02%)	855567 (4.45%)	11379 (0.06%)	18335028 (95.40%)

5

NBG-10-Month-3	16093998	0.805G	0.01%	99.55%	98.81%	45.30%	46 (0.00%)	7194 (0.04%)	849 (0.01%)	199451 (1.24%)	2304 (0.01%)	15884154 (98.70%)
LLG-1-Month-1	14643268	0.732G	0.01%	99.65%	99.08%	45.62%	2 (0.00%)	6481 (0.04%)	512 (0.00%)	69513 (0.47%)	5785 (0.04%)	14560975 (99.44%)
LLG-1-Month-2	12862816	0.643G	0.00%	99.79%	99.45%	45.63%	4 (0.00%)	2754 (0.02%)	552 (0.00%)	117996 (0.92%)	8004 (0.06%)	12733506 (98.99%)
LLG-1-Month-3	11793428	0.590G	0.01%	99.78%	99.44%	45.62%	2 (0.00%)	2953 (0.03%)	486 (0.00%)	136407 (1.16%)	7935 (0.07%)	11645645 (98.75%)
LLG-10-Month-1	18309239	0.915G	0.01%	99.74%	99.31%	47.63%	20 (0.00%)	6790 (0.04%)	888 (0.00%)	175114 (0.96%)	7499 (0.04%)	18118928 (98.96%)
LLG-10-Month-2	17695881	0.885G	0.01%	99.56%	98.85%	46.96%	43 (0.00%)	7634 (0.04%)	991 (0.01%)	195527 (1.10%)	6432 (0.04%)	17485254 (98.81%)
LLG-10-Month-3	14629434	0.731G	0.01%	99.52%	98.74%	46.34%	38 (0.00%)	7238 (0.05%)	754 (0.01%)	184389 (1.26%)	6273 (0.04%)	14430742 (98.6%)

Table S4 Statistical analysis of the Small RNAs Libraries.

Types	NBG-1-Month-1	NBG-1-Month-2	NBG-1-Month-3	NBG-10-Month-1	NBG-10-Month-2	NBG-10-Month-3	LLG-1-Month-1	LLG-1-Month-2	LLG-1-Month-3	LLG-10-Month-1	LLG-10-Month-2	LLG-10-Month-3
total	100.00%	100.00%	100.00%	100.00%	100.00%	100.00%	100.00%	100.00%	100.00%	100.00%	100.00%	100.00%
known_miRNA	59.55%	70.43%	63.75%	57.41%	57.97%	54.89%	66.62%	68.62%	65.19%	67.09%	68.13%	61.54%
rRNA	3.96%	2.36%	2.45%	3.03%	1.32%	1.50%	1.80%	1.76%	2.20%	3.25%	2.01%	1.50%

6

tRNA	1.60%	0.50%	0.43%	2.20%	0.90%	0.37%	0.67%	0.88%	0.42%	0.94%	1.33%	2.68%
snRNA	0.06%	0.02%	0.03%	0.02%	0.01%	0.01%	0.01%	0.01%	0.02%	0.01%	0.01%	0.01%
snoRNA	0.69%	0.23%	0.31%	0.98%	1.37%	0.22%	0.34%	0.35%	0.33%	0.66%	0.81%	0.58%
repeat	0.25%	0.20%	0.18%	0.20%	0.11%	0.09%	0.19%	0.16%	0.18%	0.22%	0.16%	0.13%
novel_miRNA	0.02%	0.01%	0.01%	0.02%	0.01%	0.01%	0.01%	0.01%	0.01%	0.07%	0.06%	0.03%
exon: +	1.14%	0.73%	0.57%	0.62%	0.27%	0.15%	0.56%	0.48%	0.46%	0.66%	0.39%	0.32%
exon: -	0.04%	0.02%	0.02%	0.03%	0.02%	0.01%	0.02%	0.02%	0.02%	0.04%	0.03%	0.03%
intron: +	0.96%	0.64%	0.61%	0.78%	0.53%	0.42%	0.70%	0.68%	0.76%	0.95%	0.81%	0.67%
intron: -	0.04%	0.04%	0.02%	0.03%	0.02%	0.01%	0.03%	0.03%	0.03%	0.05%	0.04%	0.04%
other	31.71%	24.82%	31.62%	34.68%	37.47%	42.31%	29.05%	26.99%	30.36%	26.07%	26.23%	32.47%

7

Table S5 Primers used for RT-qPCR in this study.

Genes	Forward: 5’-3’	Reverse: 5’-3’	Length (bp)
ACTC1	AGGCCAACCGTGAGAAGATG	GTGGTACGGCCAGAAGCATA	109
DNAJB1	ACCAAAATCACCTTCCCCAA	ATATTGTGTGGCTTGTCCTT	92
ASB5	TTCCTTCCCCTACGCACGAG	TGGTCAACATCTACACCCCAG	85
HSPA6	AACGTGCTCATTTTCGACCT	TGTTCCTGCTCAAATCCTTCCC	190
NRK	TGTCCTTCTACGGTGCAT	CTGCACATAACTCCATCACCA	81
METTL7B	GCTGGTGTTTCTCCTGACCC	CTCCATCACTCGGTTGCTCT	127
PAX7	CTGCCTAATCACATCCGCCACA	GATGGACCCAGTCTCCTGGTA	138
GAPDH	CCCGTTCGACAGATAGCCGTA	AATCCGTTCACTCCGACCT	83
U6-F	CGCTTCGGCAGCACATATAC	TTCACGAATTTGCGTGTCAT	20
miR-3958-3p	AGAUAUUGCACGGUUGAUCUCU		22
miR-136-3P	AUCAUCGUCUCAAAUGAGUCU		21
miR-495-3p	AAACAAACAUGGUGCACUUCUU		22
miR-487b-3p	AAUCGUACAGGGUCAUCCACUU		22
miR-3959-5p	GGUUGAUCAGAGAACAUACAUU		22
miR-493-3p	UGAAGGUCUACUGUGUGCCAGG		22
miR-127-3p	UCGGAUCCGUCUGAGCUUGG		20
miR-381	UAUACAAGGGCAAGCUCUCUGU		22
miR-411a-5p	AUAGUAGACCGUAUAGCGUAC		21
miR-369-3p	AAUAAUACAUGGUUGAUCUUU		21

Table S6 List of co-DE miRNAs between 1-month-old and 10-month-old Longlin and Nubian goat.

Items	miRNA	up/down
1	novel_43	down
2	novel_181	down
3	novel_140	down
4	novel_128	down
5	novel_109	down
6	chi-miR-656	down
7	chi-miR-655	down
8	chi-miR-542-5p	down
9	chi-miR-542-3p	down
10	chi-miR-495-3p	down
11	chi-miR-494	down
12	chi-miR-493-5p	down
13	chi-miR-490	down
14	chi-miR-487b-3p	down
15	chi-miR-450-5p	down
16	chi-miR-433	down
17	chi-miR-424-5p	down
18	chi-miR-412-5p	down
19	chi-miR-411b-5p	down
20	chi-miR-411a-5p	down

9

21	chi-miR-410-3p	down
22	chi-miR-409-5p	down
23	chi-miR-3959-5p	down
24	chi-miR-3958-3p	down
25	chi-miR-3955-5p	down
26	chi-miR-3955-3p	down
27	chi-miR-382-5p	down
28	chi-miR-381	down
29	chi-miR-380-5p	down
30	chi-miR-380-3p	down
31	chi-miR-377	down
32	chi-miR-376e-3p	down
33	chi-miR-376c-3p	down
34	chi-miR-376b-5p	down
35	chi-miR-376b-3p	down
36	chi-miR-376a	down
37	chi-miR-369-5p	down
38	chi-miR-369-3p	down
39	chi-miR-335-5p	down
40	chi-miR-329b-3p	down
41	chi-miR-329a-3p	down

10

42	chi-miR-323a-3p	down
43	chi-miR-19b-3p	down
44	chi-miR-199b-5p	down
45	chi-miR-199a-5p	down
46	chi-miR-181c-5p	down
47	chi-miR-154a-3p	down
48	chi-miR-144-5p	down
49	chi-miR-136-5p	down
50	chi-miR-136-3p	down
51	chi-miR-130a-3p	down
52	chi-miR-129-3p	down
53	chi-miR-127-5p	down
54	chi-miR-127-3p	down
55	chi-miR-1185-3p	down
56	chi-miR-504	up
57	chi-miR-502a	up
58	chi-miR-33b-3p	up
59	chi-miR-29b-5p	up
60	chi-miR-2411-5p	up
61	chi-miR-22-5p	up
62	chi-miR-22-3p	up

11

63	chi-miR-193b-3p	up
64	chi-miR-1468-5p	up
65	chi-miR-1388-5p	up
66	chi-miR-1388-3p	up
67	chi-miR-1307-5p	up
68	chi-let-7b-3p	up

12
